# The Vulnerable Vascular Network: Endothelial Dysfunction as a Central Driver of Intestinal Inflammation—A Systematic Review

**DOI:** 10.3390/biomedicines13112690

**Published:** 2025-11-01

**Authors:** Felicia Mihăiluță, Teodor Paul Chioașcă, Andreea Onofrei (Popa), Cristina Chelmu Vodă, Alexia Anastasia Ștefania Baltă, Oana Cristina Arghir, Doina Carina Voinescu

**Affiliations:** 1 Faculty of Medicine and Pharmacy, “Dunarea de Jos” University, 800008 Galati, Romania; felicia.mihailuta@yahoo.com (F.M.); teochioasca@gmail.com (T.P.C.); andreea.onofrei@ugal.ro (A.O.); alexiabalta@yahoo.com (A.A.Ș.B.); carinavoinescu@gmail.com (D.C.V.); 2 Research Centre in the Medical-Pharmaceutical Field, “Dunarea de Jos” University, 800010 Galati, Romania; 3 Faculty of Medicine and Pharmacy, Ovidius University, 900470 Constanța, Romania; arghir_oana@yahoo.com

**Keywords:** endothelial dysfunction, inflammatory bowel disease, gut-vascular barrier, nitric oxide, oxidative stress, angiogenesis, therapeutic targets

## Abstract

Inflammatory bowel diseases (IBDs) encompass Crohn’s disease and ulcerative colitis. They represent idiopathic and chronic inflammatory conditions. Mucosal immune dysfunction and compromised gastrointestinal barrier integrity are implicated in the pathophysiological mechanisms of inflammatory bowel diseases. Recent studies have identified endothelial dysfunction as a pivotal mediator in IBD pathogenesis. Through multiple cellular and molecular interactions, endothelial dysfunction orchestrates inflammatory responses. **Objectives:** This systematic review examines contemporary evidence (2019–2025), emphasising the role of endothelial dysfunction in intestinal inflammation mechanisms, focusing on vascular-epithelial crosstalk, molecular signalling pathways, and therapeutic implications. **Methods and results:** A comprehensive literature search was conducted using PubMed, Google Scholar, Europe PMC and DOAJ databases, focusing on peer-reviewed articles published between 2019 and 2025. Following the database search and screening process, a total of 53 studies met the eligible criteria and were included in the final analysis. Keywords included “endothelial dysfunction,” “inflammatory bowel disease,” “gut-vascular barrier,” “nitric oxide,” and “intestinal inflammation.” Contemporary research demonstrates that endothelial dysfunction in IBD manifests through decreased nitric oxide bioavailability, enhanced oxidative stress, aberrant cytokine networks, pathological angiogenesis, and compromised gut-vascular barrier integrity. The emerging concept of dual barrier dysfunction highlights the interdependent relationship between epithelial and endothelial barriers in maintaining intestinal homeostasis. **Conclusions**: Offering novel therapeutic targets for precision medicine approaches, endothelial dysfunction represents a central driver in the pathophysiological mechanism in IBD. Understanding vascular-epithelial interactions provides fundamental insights for developing targeted interventions to restore intestinal barrier function and resolve chronic inflammation.

## 1. Introduction

In industrialised countries, the global prevalence of inflammatory bowel diseases continues to increase, constituting a spectrum of gastrointestinal chronic, relapsing, and idiopathic inflammatory disorders [1,2]. A complex interplay between genetic predisposition, environmental factors, lifestyle, altered immune responses, and compromised intestinal barrier function are part of the complex and still incompletely elucidated pathogenesis of inflammatory bowel diseases [3]. According to traditional paradigms, epithelial barrier dysfunction is the main pathogenic mechanism. At the same time, emerging evidence has highlighted the critical role of endothelial dysfunction in the organisation and perpetuation of intestinal inflammation [4,5].

Regulation of vascular permeability, leukocyte recruitment, and inflammatory release is achieved through the intestinal microvascular endothelium. It serves as a dynamic interface between the systemic circulation and the local tissue environment [6]. Intestinal endothelial cells exhibit unique phenotypic characteristics that facilitate specialised functions in maintaining intestinal homeostasis, although, under inflammatory conditions, they undergo pathological activation, resulting in compromised barrier function, increased leukocyte adhesion, and sustained inflammatory responses, in contrast to other cells [7,8].

To understand the pathophysiological mechanism of the intestine, the concept of the intestinal vascular barrier (IVB) emerged [9,10]. This concept was first characterized by Spadoni et al. [11], who described a gut-vascular barrier that regulates bacterial translocation into systemic circulation. This structured endothelial barrier lines the blood and lymphatic vessels positioned beneath the epithelial layer, ensuring vascular integrity and regulating communication between immune and epithelial compartments [12]. A critical pathogenic event in inflammatory bowel disease is the disruption of the IVB. This phenomenon happens because it facilitates the transition from localised mucosal inflammation to systemic inflammatory responses [13,14].

Impaired regulation of nitric oxide signalling, oxidative stress, inflammatory cytokine networks, and pathological angiogenesis are some of the multiple molecular mechanisms underlying endothelial dysfunction in IBD [1,7,15]. The chronic nature of IBD and its resistance to classical and conventional therapeutic approaches is reinforced by these interconnected pathways, which create a self-perpetuating cycle of inflammation and tissue damage [16,17].

In addition to their impact on the gastrointestinal tract, inflammatory bowel diseases, such as ulcerative colitis and Crohn’s disease, are increasingly associated with systemic manifestations, particularly an elevated risk of cardiovascular disease [5,7]. Epidemiological studies have shown that patients with IBD have a higher incidence of ischemic heart disease, cerebrovascular events, and thromboembolic complications compared to the general population [5]. This increased cardiovascular risk cannot be fully explained by traditional risk factors (such as smoking, hypertension, or dyslipidemia), suggesting the involvement of disease-specific mechanisms, including chronic systemic inflammation, oxidative stress, endothelial dysfunction, and immune dysregulation [5,6]. Pro-inflammatory cytokines such as TNF-α and IL-6, which are frequently elevated in IBD, play a major role in endothelial damage and the progression of atherosclerosis. This is reflected in the impairment of vascular markers such as flow-mediated dilation (FMD), increased arterial stiffness (PWV, AIx), and carotid intima-media thickness (cIMT) [5,6,17]. Therefore, IBD should be regarded as systemic conditions rather than merely digestive disorders, requiring a comprehensive therapeutic approach that also addresses cardiovascular risk [7,18].

The fundamental aim of this systematic review is to determine the association between inflammatory bowel disease and endothelial dysfunction by synthesising recent evidence on circulating oxidative stress biomarkers and vascular parameters, compared to healthy controls, with a focus on the molecular pathways involved, therapeutic implications and epithelial-endothelial barrier interactions.

## 2. Materials and Methods

**Figure 1:**
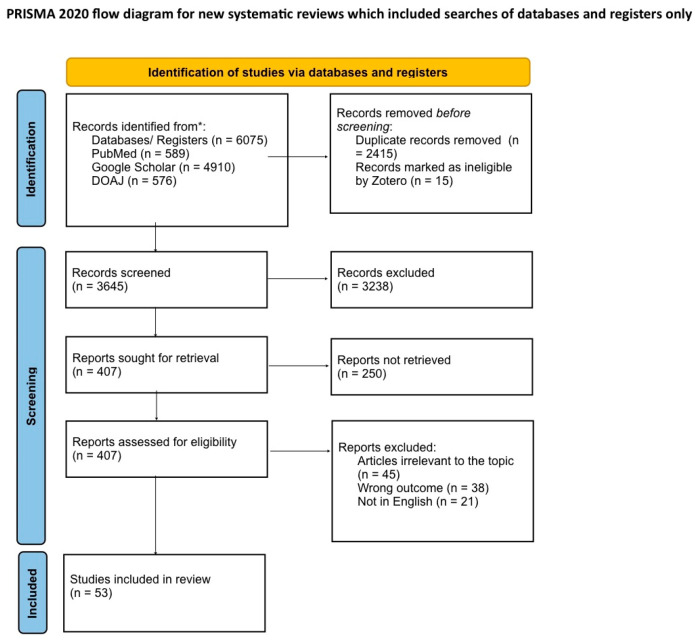
The PRISMA flow diagram. The diagram details the four-phase study selection process: identification, screening, eligibility and inclusion. From the initial 6075 records identified to the final 53 studies included in the analysis. * PRISMA: Preferred Reporting Items for Systematic Review and Meta-Analyses [19].

This systematic review was conducted according to the PRISMA 2020 reporting guidelines [19] (Figure 1) and focused on literature published between January 2019 and July 2025. The review protocol was registered in the International Prospective Register of Systematic Reviews (PROSPERO) under the registration number CRD420251134662.

Electronic databases, including PubMed, Google Scholar and DOAJ were systematically searched using predefined search strategies. The main search terms included combinations of “endothelial dysfunction”, “inflammatory bowel disease”, “Crohn’s disease”, “ulcerative colitis”, “intestinal vascular barrier”, “nitric oxide”, “oxidative stress” and “intestinal inflammation”.

The criteria for inclusion are original research articles, systematic reviews and meta-analyses published in English, focusing on the mechanisms of endothelial dysfunction in inflammatory bowel disease. Studies were selected based on their relevance to understanding the vascular pathophysiology of intestinal inflammation, with a focus on molecular mechanisms, clinical correlates and therapeutic implications. Exclusion criteria included studies published before 2019 and research not directly related to the pathophysiology of IBD. Quality assessment was performed using appropriate tools for different types of studies, and the results were synthesised to provide comprehensive information on endothelial dysfunction in inflammatory bowel diseases.

In the initial phase of the selection process, a total of 6075 articles were identified by searching three electronic databases: PubMed (*n* = 589), Google Scholar (*n* = 4910) and DOAJ (*n* = 576). After the initial collection, 2415 duplicate records were removed and another 15 articles were marked as ineligible by the bibliographic management software Zotero (version 6.0).

Thus, a total of 3645 articles were subjected to the initial screening process, which consisted of analysing the title and abstract. This led to 3238 studies that were excluded. For the remaining 407 articles, an attempt was made in order to obtain the full text. It was not possible to retrieve 250 of these reports. Therefore, 157 articles were assessed in detail for eligibility. In this final phase, 104 studies were excluded for the following reasons: 45 articles were considered irrelevant to the research topic, 38 measured parameters other than those of interest, and 21 articles were not published in English.

Finally, 53 studies met all eligibility criteria and were included in this qualitative analysis. The study selection process is detailed in the PRISMA diagram in Figure 1.

## 3. Results

### 3.1. Nitric Oxide Pathway Dysfunction

The nitric oxide pathway represents a fundamental mechanism of endothelial dysfunction in inflammatory bowel disease. It has profound implications in vascular homeostasis and inflammatory responses [20,21]. A characteristic of endothelial dysfunction is the reduction in nitric oxide production due to oxidative stress and the action of reactive oxygen species, which leads to impaired vasodilation and a pro-inflammatory endothelial phenotype.

Contemporary research has elucidated the complex mechanism of altered regulation of NO signalling in intestinal inflammation, characterised by decreased endothelial nitric oxide synthase (NOS) expression, resulting in abnormally low nitric oxide levels, under physiological conditions, but excessive nitric oxide synthesis during inflammation [22,23]. This dysregulation not only underscores the dual role of nitric oxide in maintaining vascular homeostasis versus promoting inflammation but also exemplifies how localized intestinal disease can have systemic vascular implications. Cases that reveal severe vascular involvement in diseases seemingly confined to a single organ highlight the complexity of inflammatory mechanisms and the need for a multidisciplinary approach in understanding and managing these conditions [23].

Studies has demonstrated that intestinal microvascular endothelial cells of patients with IBD exhibit significantly increased arginase activity, which competes for the common substrate L-arginine with nitric oxide synthase [20]. This enzymatic competition results in reduced nitric oxide bioavailability and compromised endothelial function. Under normal physiological conditions, eNOS catalyses the conversion of L-arginine to nitric oxide and L-citrulline, using tetrahydrobiopterin (BH4) as an essential cofactor. This process maintains endothelial function by regulating vascular tone, inhibiting platelet aggregation, and modulating inflammatory cell adhesion [20].

The RhoA/ROCK pathway appears as a critical negative regulator of nitric oxide generation, along with TNF-α and lipopolysaccharide-induced activation, thus leading to increased arginase expression and reduced eNOS activity [20].

In intestinal inflammation, oxidative stress leads to the uncoupling of eNOS. In this case, the enzyme produces superoxide anion (O_2_^−^) instead of nitric oxide. This phenomenon occurs through multiple mechanisms. Some of these are: oxidative depletion of the critical cofactor BH4, substrate deficiency of L-arginine and accumulation of asymmetric dimethylarginine (ADMA), an endogenous inhibitor of eNOS. The resulting reduction in NO bioavailability compromises endothelium-dependent vasodilation and promotes inflammatory activation. As we knew, the intestinal microbiota plays a crucial role in NO homeostasis; however, recent studies have revealed that this is due to the enterosalivary nitrate-nitrite-nitric oxide pathway. Therefore, the dysbiosis associated with Crohn’s disease and ulcerative colitis disrupts this pathway. Thus, it leads to decreased NO production and increased inflammatory susceptibility. In addition, excessive NO production during inflammatory states can paradoxically contribute to tissue damage through the formation of peroxynitrite and other reactive nitrogen species [15].

From the standpoint of clinical relevance, impaired physiological vasodilation, compromised tissue perfusion, and increased recruitment of inflammatory cells are manifestations of altered nitric oxide signalling in IBD [24,25]. Meta-analytic data show that patients with IBD have significantly reduced flow-mediated dilation compared with healthy controls (Cohen’s d: −0.73; 95% CI: −1.10, −0.36), indicating significant endothelial dysfunction [26].

A common and consistent feature in both Crohn’s disease and ulcerative colitis has been the alteration of the L-arginine/nitric oxide pathway. This has been demonstrated by transcriptomic analyses of the RNA transcript set and metabolomic analyses of all metabolites [15]. These studies demonstrate a symmetry in inflammatory bowel diseases of low levels of arginine and dimethyl-arginine, along with increased concentrations of citrulline and dimethylamine, reflecting the alteration of inflammatory pathways and their burden [15]. Recent evidence highlights that endothelial injury and microvascular alterations directly contribute to intestinal inflammation and oxidative imbalance [4].

### 3.2. Oxidative Stress and Reactive Oxygen Species

Regarding the molecular mechanisms of oxidative damage, along with compromised antioxidant defence systems characterised by excessive production of reactive oxygen species (ROS) and reactive nitrogen species (RNS), oxidative stress is the central pathogenic mechanism in inflammatory bowel diseases [1,12,27]. According to the most recent research, intestinal inflammation generates a complex environment of oxidative stress factors which directly affect endothelial function and perpetuate inflammatory responses [28,29]. Also, recent studies emphasize the role of the gut-vascular barrier in maintaining intestinal homeostasis and preventing systemic dissemination of inflammatory mediators [9]. Bacterial translocation has also been proposed as a driver of endothelial inflammation and systemic oxidative stress in IBD [10].

Recent clinical studies have demonstrated the therapeutic potential of oxidative stress intervention in inflammatory bowel disease (IBD) [28,30,31]. Exogenous stressors, including environmental pollutants and particulate matter are known to amplify oxidative stress and compromise the integrity of intestinal barrier. This process establishes a critical link between environmental exposures and the onset of endothelial dysfunction [30]. Moreover, oxidative stress has been shown to be a pivotal mediator in the pathogenic pathway between endothelial dysfunction and IBD, underscoring redox dysregulation as a viable therapeutic objective [1]. In experimental models of colitis, selenium-containing amino acids, including selenocysteine and selenocystine, have demonstrated significant protective effects by reducing ROS production, increasing antioxidant enzyme activity, and suppressing the expression of pro-inflammatory cytokines, particularly TNF-α and IL-6 [31].

Natural antioxidant compounds show promising therapeutic potential. This happens due to their ability to scavenge ROS and provide antioxidant defence [1]. Multi-targeted anti-inflammatory have been demonstrated to be effective by polyphenolic substances, including resveratrol, turmeric, and quercetin, through modulation of the NF-κB and Nrf2 signalling pathways [1,32]. Mitochondrial impairment, coupled with the augmented generation of reactive oxygen species is known to exacerbate endothelial stress and promote apoptosis [24,25].

### 3.3. Inflammatory Cytokine Networks

Studies consistently show elevated TNF-α, IL-6 and IL-1β expression in intestinal and vascular tissue of IBD patients. Vascular activation and remodeling have also been recognized as key features of endothelial dysfunction induced by cytokines in IBD [5]. The inflammatory cytokine network is a key mechanism driving endothelial dysfunction in inflammatory bowel diseases, as the intestinal endothelium serves as both a target and a source of inflammatory mediators. The main mediators of vascular pathology are: TNF-α, IL-1β and IL-6. TNF-α, by activating NF-κB signalling, increasing ROS production and up-regulating adhesion molecules, including ICAM-1 (Intercellular Adhesion Molecule-1) and VCAM-1 (Vascular Cell Adhesion Molecule-1), induces endothelial dysfunction. This cytokine promotes endothelial permeability by disrupting VE-cadherin junctions and reorganisation of the actin cytoskeleton [5,30]. Recent studies have shown that IL-6 trans-signaling induces endothelial activation via plasminogen activator inhibitor-1 secretion [33], while multi-omics analyses defined distinct endothelial inflammatory states induced by citokines [34,35]. Other reserch identified IFN-γ as a significant driver of sustained endothelial costimulatory phenotype [36], with downstream effects on MAPK pathways and apoptosis regulation [37]. Also, emerging protein interaction studies highlight novel mediators: TRAF6, which further contribute to endothelial activation and inflammatory signalling [36].

These cumulative findings have revealed the complex molecular mechanisms underlying endothelial inflammatory states.

Underscoring the role of cytokine interactions in pathophysiological processes, combined stimulation with TNF-α and IFN-γ induces synergistic inflammatory processes in endothelial cells. The transcription factors AP-1 and NF-κB, through the activation of MAP kinase, are stimulated by these cytokines. Consequently, they lead to increased expression of adhesion molecules, chemokines, and pro-inflammatory mediators.

Endothelial cells function not only as targets of inflammatory cytokines, but also as active producers of inflammatory mediators. Human endothelial cells express multiple cytokines, including IL-1, IL-6, IL-8, IL-15, and various colony-stimulating factors. This dual role creates an amplification loop that sustains chronic inflammatory responses in inflammatory bowel disease.

Recent studies demonstrate that adhesive interactions between endothelial cells and recruited inflammatory cells signal the secretion of additional inflammatory cytokines, creating critical crosstalk mechanisms for chronic inflammatory states. This endothelial-immune cell–cell communication represents a potential therapeutic target for disrupting inflammatory cascades.

### 3.4. Pathological Angiogenesis and VEGF Alteration

Vascular endothelial growth factor (VEGF) alteration is a hallmark of the pathophysiology of inflammatory bowel diseases. Systematic reviews and meta-analyses confirm elevated circulating VEGF levels in patients with inflammatory bowel diseases [2,38]. By having a dual role, VEGF promotes the potential of tissue repair while simultaneously supporting inflammatory responses through increased vascular permeability and immune cell recruitment [16,39].

The corticotropin-releasing hormone (CRH) family of peptides has been identified as regulators of VEGF-A production in intestinal epithelial cells. The process happens through activation of the cAMP/CREB pathway [40,41]. The neuroendocrine-angiogenic axis represents a novel mechanism. It links stress responses to intestinal inflammation and vascular dysfunction [40].

Studies using VEGF-C over-expression in experimental models of colitis demonstrate increased epithelial damage, inflammatory oedema, and neutrophil infiltration, with inflammatory lymphangiogenesis potentially exacerbating intestinal inflammation [39,41,42]. Thus, targeting pathological angiogenesis in IBD may represent a therapeutic strategy to consider [14,16]. Proteins related to angiogenesis with potential as therapeutic targets include hypoxia-inducible factor, angiopoietins, and basic fibroblast growth factor [16]. Anti-TNF-α antibodies demonstrate efficacy in part by down-regulating intestinal angiogenesis, contributing to their therapeutic success [16]. Experimental models further demonstrate that VEGF-targeted therapies can modulate angiogenic signaling and reverse endothelial activation [42]. Endothelial dysfunction not only causes intestinal inflammation, but also increases cardiovascular risk observed in IBD patients [13,17]. This link between intestinal and systemic vascular alterations is reinforced by evidence implicating gut microbiome dysregulation in endothelial activation and cardiovascular comorbidities [17].

### 3.5. Alteration of Adhesion Molecules

The molecular mechanisms of leukocyte recruitment occur through the up-regulation of adhesion molecules. This represents a critical mechanism of endothelial dysfunction in inflammatory bowel diseases, facilitating leukocyte recruitment and increased tissue infiltration [43,44]. During inflammatory states, ICAM-1, VCAM-1, and E-selectin are significantly increased on the intestinal microvascular endothelium [45,46].

By increasing the expression and function of endothelial P-selectin, E-selectin, and VCAM-1, TNF-α promotes leukocyte rolling and adhesive interactions [44]. This mechanism directly correlates with increased microvascular injury and tissue damage [44]. Furthermore, microbiota-immune interactions have been shown to regulate adhesion molecule expression, reinforcing the gut barrier’s role in vascular homeostasis [3].

From the standpoint of targeted therapy at the level of adhesion molecules, blocking trafficking molecules has emerged as a therapeutic innovation for IBD [47,48]. Natalizumab, Vedolizumab, and Etrollizumab are examples of successful anti-integrin therapies, while MAdCAM-1 and ICAM-1 inhibitors are under investigation [47]. Selective targeting of gut-specific adhesion pathways is envisioned with the goal of minimising systemic immunosuppression while simultaneously controlling intestinal inflammation [47,48]. These precision approaches offer improved safety profiles compared to systemic immunosuppressive agents [48,49].

### 3.6. Therapeutic Interventions and Future Directions

Targeting endothelial dysfunction, along with inflammatory pathways, has gained importance regarding the treatment of inflammatory bowel diseases [50,51]. Having demonstrated partial efficacy, anti-TNF therapy restores endothelial function and reduces vascular inflammation [7,52]. Management of cardiovascular risk in IBD increasingly incorporates endothelial biomarkers as therapeutic indicators [6]. Recent evidence also highlights ST2 and CSF-1 as potential endothelial therapeutic targets in IBD, linking chronic inflammation to vascular remodelling [49].

Novel therapeutic approaches include receptor-mediated drug delivery systems. These exploit specific receptors and adhesion molecules on inflamed endothelial cells [50]. These precision systems have the ability to achieve higher local drug concentrations with minimal systemic side effects [50].

Future therapeutic directions focus on multi-targeted approaches that address the complex pathophysiology of endothelial dysfunction in IBD [53,54]. Neutrophil extracellular traps (NETs) with ROS-sensitive nano-carriers represent promising approaches for precision medicine [53,54]. Neuropsychological comorbities in IBD have been linked to endothelial dysfunction through the gut–brain axis [8].

New therapeutic opportunities are represented by metabolic interventions targeting mitochondrial dysfunction and immunometabolic pathways [55]. The aim is to address the fundamental cellular energetic disturbances that underlie chronic inflammation [55].

### 3.7. Quantitative Synthesis and Forest Plot Analysis

This systematic review synthesises evidence regarding endothelial dysfunction in IBD. We focused on five mechanisms: nitric oxide dysfunction, oxidative stress, inflammatory cytokines, pathological angiogenesis and VEGF alterations. These mechanistic domains, beside reflecting mucosal inflammation, contribute to the elevation of cardiovascular burden seen in IBD patients.

**Figure 2:**
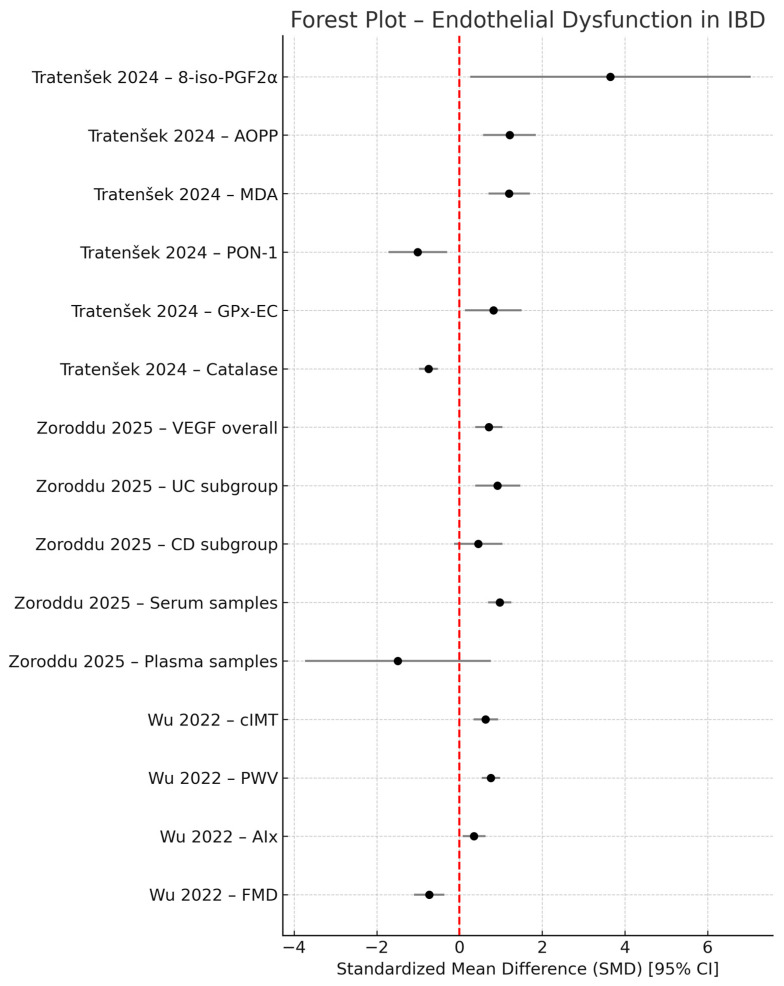
Forest plot illustrating the standardized mean differences (SMD, 95% CI) in oxidative stress, angiogenic signalling (VEGF) and vascular function metrics (cIMT, PWV, Aix, FMD) comparing patients with inflammatory bowel disease (IBD) and healthy controls [1,38,51].

The forest plot analysis offers quantitative evidence for a multifactorial model of endothelial dysfunction in IBD and the summary of standardized mean differences is illustrated in Figure 2. The synthesis shows convergent evidence for endothelial alteration by combining biochemical markers, angiogenetic factors and vascular function measures. The forest plot analysis standardised mean differences (SMD, 95% CI) by comparing IBD with healthy controls across three parameters: oxidative stress biomarkers, angiogenetic signalling (VEGF) and vascular function metrics (cIMT, PWV, AIx, FMD). By convention, SMD > 0 shows higher values in IBD, SMD < 0 shows lower values in IBD and CIs excluding 0 shows statistical significance.

Markers of oxidative damage are consistently high with moderate to large effects: (MDA, SMD~1.20, 95% CI 0.70–1.70) and advanced oxidation protein (AOPP, SMD~1.21, 0.57–1.85). 8-iso-PGF_2_α exhibit a large point estimate (SMD~3.65) but a wide Ci (0.26–7.04) showing imprecision and heterogeneity rather than a uniformity effect. Antioxidant defences are reduced, with PON-1 (SMD~−1.01, −1.72 to −0.30) and catalase (SMD~−0.75, −0.98 to −0.52) demonstrating moderate to large deficits. GPx-EC displays a smaller increase (SMD~0.82, 0.13–1.50), underscoring assay- and pathway- specific responses, not necessarily a uniformity antioxidant depression. The figure reveals a directionally coherent redox shift toward oxidative injury in IBD.

Angiogenic signalling (VEGF) levels were consistently elevated overall and within disease subtype. Subgroup estimates showed disease and matrix dependent heterogeneity, with a moderate increase in ulcerative colitis (SMD = 0.92, 95% CI 0.38–1.47), a smaller increase in Crohn’s disease (SMS = 0.45, 95% CI 0.13–1.04) and a significant increase in serum samples (SMD = 0.97, 95%CI 0.69–1.25), while plasma estimates were non-significant. These results confirm enhanced pro-angiogenetic signalling in IBD [38].

The vascular parameters cIMT and PWV were significantly increased in IBD patients, indicating arterial sclerosis, while Aix showed a smaller increase. Flow-mediated dilation (FMD) was decreased, showing impaired NO-dependent vasodilation and confirming endothelial dysfunction [51].

Wide confidence intervals (CIs) reflect patient and study heterogeneity and methodological imprecision. Due to the fact that all effects are expressed as standardized mean differences (SMDs), differences in measurement units and assays are normalized. However, matrix type, disease subtype and methodology explain the few non-significant estimates.

## 4. Discussion

### 4.1. Pathophysiological Integration

The quantitative synthesis confirmed the multifactorial nature of endothelial dysfunction in inflammatory bowel disease, integrating oxidative stress, angiogenic signalling and vascular alterations. The current understanding of endothelial dysfunction in inflammatory bowel disease reveals a complex, interconnected network of pathological processes that collectively lead to chronic intestinal inflammation. The evidence presented demonstrates that endothelial dysfunction is not simply a consequence of inflammation, but rather a central pathogenic mechanism that orchestrates and perpetuates inflammatory responses through multiple molecular pathways.

Fundamentally, alterations in nitric oxide signalling underlie endothelial dysfunction in IBD. Competitive inhibition of eNOS causes a pathological switch from protective NO signalling to the production of inflammatory mediators [15,20]. Compromised vascular homeostasis and increased recruitment of inflammatory cells are due to this metabolic reprogramming. Thus, a self-perpetuating cycle of endothelial dysfunction and tissue damage is established.

Oxidative stress is another critical pathogenic mechanism. Excessive production of ROS/RNS overwhelms antioxidant defence systems and causes direct endothelial damage [1,29]. Amplification loops are created. They sustain chronic inflammation and prevent the resolution of tissue damage. Thus, the therapeutic potential of antioxidant interventions has been shown. This validates the importance of targeting oxidative stress in the management of inflammatory bowel diseases [28,31].

Current therapies used in inflammatory bowel diseases influence not only local inflammation but also systemic vascular function. By reducing chronic inflammatory processes, these treatments may help improve endothelial dysfunction, arterial stiffness, and oxidative stress—factors involved in the increased cardiovascular risk associated with these conditions. Certain anti-inflammatory therapies have demonstrated the ability to reduce endothelial adhesion molecule expression and vascular permeability, thereby contributing to the preservation of vascular integrity. Other therapeutic classes, with more selective action on the gastrointestinal tract, may offer a more favorable cardiovascular safety profile by limiting systemic effects. Additionally, some therapies targeting specific pro-inflammatory pathways may also impact pathological angiogenesis, with potential benefits but also risks regarding tissue healing. Therefore, a balanced therapeutic approach is needed—one that addresses both intestinal inflammation control and the maintenance of vascular function. Monitoring vascular biomarkers and endothelial function parameters may provide further insights for optimizing treatment in these conditions.

### 4.2. The Gut-Vascular Barrier Paradigm

To understand the function of the gut barrier, the concept of the gut-vascular barrier emerged, which emphasises the interdependent relationship between the epithelial and endothelial barriers in maintaining intestinal homeostasis [9,12]. In inflammatory bowel diseases, alterations in both barriers create multiple points of vulnerability. At these points, bacterial translocation, systemic inflammation, and chronic disease progression are facilitated [14,49]. The clinical implications of extend beyond local intestinal pathology, such as systemic manifestations and cardiovascular complications [13,18]. Recognition of the risk, at the systemic cardiovascular level, provides a framework for understanding the increased cardiovascular morbidity observed in populations with inflammatory bowel diseases (IBD) [14,26].

Understanding the mechanisms of endothelial dysfunction opens multiple therapeutic perspectives for precision approaches. Current therapeutic strategies, such as anti-TNF therapy and adhesion molecule blockers, demonstrate partial efficacy by restoring endothelial function [16,47]. However, the complex and multifactorial nature of the dysfunction suggests that a combined approach may be necessary to achieve optimal clinical outcomes. Emerging therapeutic strategies focus on targeting specific molecular pathways underlying endothelial dysfunction, including metabolic interventions, antioxidant therapies, and precision drug delivery systems [50,53,55]. These approaches offer the potential to address fundamental pathogenic mechanisms while minimising systemic toxicity by targeting inflamed tissues.

Ideally, biomarkers that reflect the state of endothelial dysfunction would be developed, which could facilitate personalised therapeutic approaches and monitoring of treatment responses. Metabolomic and transcriptomic signatures of endothelial dysfunction may provide valuable tools for implementing precision medicine in the management of IBD [15,49].

### 4.3. Future Research Directions

Future research should focus on elucidating the temporal connection between different aspects of endothelial dysfunction and their relative contributions to the pathogenesis of inflammatory bowel diseases. Longitudinal studies examining the evolution of endothelial dysfunction during disease progression could identify critical points of intervention to prevent chronic inflammation and tissue damage. The development of new therapeutic targets requires a deeper understanding of tissue-specific endothelial cell phenotypes and their responses to inflammatory stimuli. Single-cell transcriptomics and organ-on-chip models offer promising approaches to dissect the heterogeneity of endothelial responses across different intestinal regions and inflammatory contexts [50,55].

In recent years, increasing attention has been directed toward endothelial dysfunction as a central component in the pathogenesis of inflammatory bowel diseases, particularly due to its contribution to both local and systemic inflammatory complications. While conventional therapies have primarily targeted immune pathways, there is growing recognition of the need to integrate vascular health into IBD management.

Anti-TNF therapy, a cornerstone in IBD treatment, has shown partial success in restoring endothelial function and reducing vascular inflammation, underscoring its dual immunological and vascular impact. However, therapeutic responses remain heterogeneous, and not all patients achieve sustained remission. This highlights the necessity for more refined therapeutic strategies that can address endothelial involvement specifically [52,54].

Emerging endothelial biomarkers, such as ST2 and colony-stimulating factor 1 (CSF-1), have shown promise not only as indicators of disease activity but also as potential therapeutic targets. Their role in mediating vascular remodelling and perpetuating chronic inflammation suggests that modulating these pathways could yield vascular and intestinal benefits [5,7].

Innovative drug delivery systems, such as receptor-mediated nanoparticles targeting adhesion molecules on inflamed endothelium, represent a significant advancement in precision medicine. These systems enhance local drug concentration at the site of inflammation while minimizing systemic toxicity, thus improving both efficacy and safety profiles.

Future directions in IBD therapy are moving toward multi-targeted interventions, recognizing the interplay between immune dysregulation, oxidative stress, and endothelial dysfunction. Among these, ROS-sensitive nano-carriers, capable of releasing drugs in response to oxidative environments such as those found in inflamed tissues, represent a particularly promising strategy.

Additionally, the gut–brain axis has emerged as a critical area of exploration, particularly given the observed association between endothelial dysfunction and neuropsychological comorbidities in IBD patients. This connection supports the concept that endothelial-targeted therapies may have broader systemic effects beyond the gut.

Another promising avenue involves metabolic interventions, which aim to correct mitochondrial dysfunction and immunometabolic disturbances underlying chronic inflammation. By restoring cellular energy balance, these strategies hold the potential to not only modulate immune responses but also improve endothelial integrity.

### 4.4. Challenges of Clinical Translation

Translating endothelial dysfunction research into clinical practice faces several challenges, including the development of reliable biomarkers, standardisation of assessment methods, and integration with existing therapeutic approaches. The complexity of the mechanisms of endothelial dysfunction requires sophisticated diagnostic tools to identify patients who would benefit most from endothelium-directed therapies.

The safety profile of endothelial-targeted interventions requires careful evaluation, especially given the essential roles of endothelial cells in cardiovascular homeostasis. Clinical trials of endothelial-targeted therapies must balance efficacy in controlling intestinal inflammation with potential cardiovascular and systemic effects.

### 4.5. Biomarker Development

The progression of biomarker discovery opens a crucial pathway. It facilitates the transposition of fundamental research on endothelial dysfunction into practical clinical applications for the management of IBD. Recent studies have brought to attention new circulating biomarkers, showing the complex interplay between gut dysbiosis, increased cardiovascular risk and systemic inflammation [50]. In this context, several emerging biomarkers are proving to be promising tools: short chain fatty acids (SCFAs), zonulin and asymmetric dimethylarginine (ADMA). They offer the opportunity to stratify patients, not only according to the severity of the endothelial dysfunction, but also according to individual cardiovascular risk profiles. A paradigm shift is represented by the identification of RNA isoforms in peripheral blood. It is a non-invasive monitoring technique of intestinal barrier integrity [53]. Particularly, MUC20-OT1 variants have been proven to correlate with endoscopic inflammation scores and histological disease activity. This suggests a new way of assessing the intestinal barrier dysfunction without using invasive procedures. New research applying advanced statistical methods, has been able to identify new potential therapeutic targets: ST2 and CSF-1, which show casual association with the risk of developing IBD and could simultaneously serve as prognostic biomarkers and targets for future therapies. The integration of multi-omics approaches is about to revolutionise our understanding, revealing comprehensive molecular signatures of endothelial dysfunction [55]. Such a perspective is essential to pave the way for the real implementation of precision medicine in the management of IBD, these biomarkers holding the promise of allowing us to accurately identify those patients with IBD with higher risk of developing endothelial dysfunction and cardiovascular complications. Such early identification would enable targeted interventions, applied before vascular damage would become irreversible.

### 4.6. Limitations

This systematic review has several limitations. First, the search was restricted to three databases: PubMed, DOAJ and Google Scholar and to articles published from 2019 onwards, which may exclude relevant studies published earlier or indexed elsewhere. Secondly, only articles in English were included, which could introduce a language bias. Although, 53 studies met the eligible criteria, they were heterogeneous in methodology, study design and population characteristics, which could limit comparability. The quality of the included studies varied, some being observational and based on small group sizes. Finally, despite the meta-analytic approach, the conclusions should be interpreted with cautions, given the variability of the included literature.

We used the PRISMA checklist when drafting our report [19].

## 5. Conclusions

This systematic review demonstrates that endothelial dysfunction is a central pathophysiological mechanism in inflammatory bowel diseases. It orchestrates chronic inflammation through multiple interconnected molecular pathways. Evidence shows that intestinal endothelial cells undergo pathological activation. It is characterised by altered nitric oxide signalling, increased oxidative stress, aberrant cytokine production, pathological angiogenesis, and compromised barrier function.

The emergence of the gut-vascular barrier concept has provided a framework for understanding the interdependent relationship between epithelial and endothelial barriers in maintaining intestinal homeostasis. Dual barrier dysfunction creates multiple points of vulnerability. These points facilitate disease progression and systemic complications, providing new therapeutic targets for intervention. Contemporary therapeutic approaches increasingly recognise the importance of targeting endothelial dysfunction alongside traditional inflammatory pathways. Precision medicine strategies, such as targeted drug delivery systems, metabolic interventions, and combination therapies, offer promising prospects for addressing the complex pathophysiology of endothelial dysfunction in inflammatory bowel disease (IBD).

Future research should focus on developing personalised therapeutic approaches based on individual endothelial dysfunction profiles, establishing reliable biomarkers for monitoring treatment responses, and translating mechanistic insights into effective clinical interventions. A comprehensive understanding of the mechanisms of endothelial dysfunction provides a basis for the development of next-generation therapies that address the fundamental pathogenic processes underlying chronic intestinal inflammation.

The therapeutic implications of this research extend beyond inflammatory bowel disease, such as other chronic inflammatory conditions characterised by endothelial dysfunction. The principles and mechanisms elucidated in barrier-immune-interaction (BII) research may elucidate therapeutic approaches for a broader spectrum of inflammatory diseases, contributing to the advancement of precision medicine in inflammatory disorders.

## Data Availability

The data supporting the findings of this study are available in the cited articles and publicly accessible databases.

## Abbreviations

The following abbreviations are used in this manuscript:

IBD	Inflammatory bowel diseases
IVB	intestinal vascular barrier
eNOS	endothelial nitric oxide synthase
RhoA/ROCK	Ras homolog gene family member A/Rho-associated coiled-coil-containing protein kinase
RNA transcript	Ribonucleic acid copy transcribed from DNA
TNF-α	Tumor Necrosis Factor alpha
IL-6	Interleukin-6
IL-1β	Interleukin-1 beta
ICAM-1	Intercellular Adhesion Molecule-1
VCAM-1	Vascular Cell Adhesion Molecule-1
NF-κB	Nuclear Factor kappa-light-chain-enhancer of activated B cells
Nrf2	Nuclear factor erythroid 2–related factor 2
IFN-γ	Interferon gamma
MAPK	Mitogen-Activated Protein Kinase
TRAF6	TNF Receptor Associated Factor 6
MAP kinase	Mitogen-Activated Protein kinase
cAMP/CREB	Cyclic Adenosine Monophosphate/cAMP Response Element-Binding Protein
MAdCAM-1	Mucosal Addressin Cell Adhesion Molecule-1
ST2	Suppression of Tumorigenicity 2 (IL-33 receptor)
CSF-1	Colony-Stimulating Factor 1
Vascular function metrics	cIMT (Carotid Intima-Media Thickness), PWV (Pulse Wave Velocity), AIx (Augmentation Index), FMD (Flow-Mediated Dilation)
GPx-EC	Glutathione Peroxidase in Erythrocytes
MUC20-OT1	Mucin 20 overlapping transcript 1
